# Summer Medicine

**Published:** 1915-06

**Authors:** 


### Summer Medicine

Impress upon your patients who are in delicate health from almost any cause, the economic value of rest, plain living, out-door air. Various forms of anaemia, chronic constipation and alimentary putrefaction, leanness and obesity, catarrhal tendencies, incipient pthisis, insomnia, nervous breakdown and many other conditions may give place to health if the summer is properly used. The prosperous and the thriftless need no incentive to a vacation but the great, toiling, ambitious middle class between these extremes must be taught to regard the summer vacation as a good investment. A large proportion of the medical profession need to take this advice home to themselves. In many instances, your advice will be necessary to secure a wise choice between a long rest with simple living and a short, expensive, sojourn at a fashionable resort or a trip involving the expense and hygienic defects of much travel or hard work at some summer school. When expense and loss of time must be considered, week-end and afternoon trips, using the trolley, bicycle, excursion boat and those much-neglected means of locomotion, the legs, may furnish a very satisfactory substitute for a regular vacation.

· Do not forget that, the hygienic advantages of the country are often outweighed by sanitary defects. The larger cities have already achieved a lower death rate than the rural districts and the worst sanitary conditions are found in some villages and small cities. Typhoid, para-typhoid and colon bacillus infections are to be especially feared from contaminated wells. The late summer and autumnal increase in typhoid is largely due to infection during vacations in the country or at suburban resorts. Tetanus is, essentially, a manure-borne infection, the bacilli requiring, in the majority of cases, a hermetic sealing of the wound, such as occurs from the explosion of toy-pistol cartridges, etc., or punctured wounds. An apparently ideal, secluded country home may be just the place where the danger of tuberculosis is greatest, human bacilli being implanted from bedding infected years previously and never properly renovated, bovine bacilli from the few cattle used for domestic purposes and escaping the inspection applied more stringently to dairy farms. Various infections may be caused by flies which often swarm about privies, styes, manure piles and carcasses of fowls and domestic animals. Poetry and cherished notions should not blind us to the fact that there are many country homes in which the lack of sanitary decency and ordinary cleanliness and the indifference to the rudiments of dietetics, surpass the worst conditions of the slums and cheap restaurants of a city. The possibility of malarial and dysenteric infections, of certain rare diseases communicated from the lower animals, of fomites of small pox, diphtheria and the exanthemata, perhaps dating many years back, should be remembered. If possible, the physician should inspect the places at which his clientele expect to spend the summer; at least, general warnings should be given.

Certain forms of what may be termed clinical research, should receive the attention of the medical profession during the summer, especially by those members of the profession who practice in the country or are stationed at camps and summer resorts. The last word has not yet been written as to ivy-poisoning nor on the poisoning by ingestion of various toxic berries. The annual recurrence of ivy-poisoning, without fresh exposure, is still claimed by many. Children and their guardians should be warned as to these dangers. On the one hand, the summer affords excellent opportunities for the Rollier treatment, on the other hand the dangers, clinical manifestations and treatment of insolation and ordinary excessive sun-burn deserve careful attention. That excessive indulgence in swimming is harmful, is well established but it is not definitely known just how much of the danger is due to "water soaking" pure and simple, how much to superficial infection from contaminated water, how much to insect and leech bites, wounds poisoned by rushes, etc. Dermal lesions afford opportunities for careful study also. It may not be entirely a grandmother's notion that toads and similar animals cause warts by the irritation of specific toxins.

The botanic materia medica affords an interesting study during the summer. It is not unreasonable to suppose that the old notions of the herbalists may have a considerable basis in fact and, at any rate, many thoroughly studied vegetable drugs are indigenous to this region and, in the present state of the drug market, have a commercial value that would repay the labor of collecting them. For example, you may have in mind some school boy or girl or even an adult, who needs a vacation and cannot afford one. Such a person might make his expenses by gathering medicinal plants, with a very little educative assistance. Indeed, if he could find a neglected patch of woodland along a stream where hydrastis is common, he might make a small fortune.

Every physician should, so far as possible, resolve himself into a university extension course in summer hygiene. In particular, we would urge, a campaign against flies and equally against literal fly-swatting and the enlistment of susceptible children in too direct activities in such a campaign. Wholesale, non-manual methods of attacking flies should be employed.

Summer is a critical period, from the hygienic and sanitary standpoint. It affords the opportunity for storing up energy for the winter; it involves its own dangers, largely because it is the period at which microscopic crops of bacteria, fungi and animal organisms directly concerned with disease production or indirectly but inevitably concerned with the conveyance of disease germs, grow even more luxuriantly and through many more generations, than do macroscopic crops.

